# Molecular Cloning, Characterization and Expression Profiling of a Ryanodine Receptor Gene in Asian Corn Borer, *Ostrinia furnacalis* (Guenée)

**DOI:** 10.1371/journal.pone.0075825

**Published:** 2013-10-01

**Authors:** Li Cui, Daibin Yang, Xiaojing Yan, Changhui Rui, Zhenying Wang, Huizhu Yuan

**Affiliations:** Key Laboratory of Integrated Pest Management in Crops, Ministry of Agriculture, Institute of Plant Protection, Chinese Academy of Agricultural Sciences, Beijing, China; Swedish University of Agricultural Sciences, Sweden

## Abstract

Ryanodine receptor (RyR) Ca^2+^ release channel is the target of diamide insecticides, which show selective insecticidal activity against lepidopterous insects. To study the molecular mechanisms underlying the species-specific action of diamide insecticides, we have cloned and characterized the entire cDNA sequence of RyR from *Ostrinia furnacalis* (named as *Of*RyR). The *Of*RyR mRNA has an Open Reading Frame of 15324 bp nucleotides and encodes a 5108 amino acid polypeptide that displays 79–97% identity with other insects RyR proteins and shows the greatest identity with *Cnaphalocrocis medinalis* RyR (97%). Quantitative real-time PCR showed that the *Of*RyR was expressed at the lowest level in egg and the highest level in adult. The relative expression level of *Of*RyR in first, third and fifth-instar larva were 1.28, 1.19 and 1.99 times of that in egg. Moreover, two alternative splicing sites were identified in the *Of*RyR gene. One pair of mutually exclusive exons (a/b) were present in the central part of the predicted SPRY domain, and an optional exon (c) was located between the third and fourth RyR domains. Diagnostic PCR demonstrated that exons a and b existed in all developmental stages of *Of*RyR cDNA, but exon c was not detected in the egg cDNA. And the usage frequencies of these exons showed a significant difference between different developmental stages. These results provided the crucial basis for the functional expression of *Of*RyR and for the discovery of compound with potentially selective insect activtity.

## Introduction

Ryanodine receptors (RyRs) are members of a superfamily of intracellular Ca^2+^ channels. These channels regulate the release of calcium from the lumen of sarcoplasmic/endoplasmic reticulum to the cytosol of muscle and non-muscle cells [1]–[2]. The RyRs are homomeric tetramers with a total molecular mass of 2 to 2.5 MDa, and each contains about 5000 amino acids with a large hydrophilic N-terminal domain and a small membrane-spanning C-terminal domain. The large N-terminal cytoplasmic domain termed “foot structure” spans the junction gap between the transverse tubules and sarcoplasmic reticulum membranes, while the hydrophobic C-terminal domain, which was predicted to contain 4–12 transmembrane segments, forms the putative pore of the Ca^2+^ release channel [3]–[4]. It has been proposed that the specific interaction between the cytoplasmic and transmembrane domains is an important mechanism in the intrinsic modulation of the RyR [5].

To date, three types of ryanodine receptor (RyR1, RyR2 and RyR3) have been identified in mammals. RyR1 and RyR2 are abundant in skeletal and cardiac muscles. RyR3 is ubiquitously expressed throughout different tissues but is relatively abundant in brain and certain skeletal tissues [6]–[8]. There is approximately 66% amino acid sequence homology among the three mammalian RyR isoforms, except for three regions with high degrees of variability [9]. Two isoforms (RyRA and RyRB) were found in fish, amphibians and birds [10]–[11]. These two isoforms showed homology with mammals RyR1 and RyR3. However, only one RyR gene was found in *Drosophila melanogaster* (*Dm*RyR) [12], and *Dm*RyR shared 45–47% amino acid identity with three mammalian RyR isoforms [13]. The regions with a high degree of structural divergence between the mammalian and insect isoforms of the RyR may serve as potential targets for insecticides that interact specifically with the insect but not mammalian isoforms [9].

The Ca^2+^-release channel properties of RyR have been studied with increasing interest in the last 30 years in the medical field. Recently, insect RyRs attracted researchers' great attention in the pesticide field. Because, insect RyRs were found to be the targets of two classes of diamides chemicals: the phthalic diamides such as flubendiamide [14]–[17], and the anthranilic diamides such as chloranthraniliprole [18]–[20]. The diamides insecticides activate the ryanodine-sensitive intracellular calcium release channels in insects and show good selectivity for insect RyRs compared to mammalian RyRs by evoking typical symptoms including poisoning, body contraction, feeding cessation, paralysis and subsequent mortality [21]. It was reported that flubendiamide and chlorantraniliprole caused release of intracellular Ca^2+^ in isolated *Heliothis virescens* and *Periplaneta americana* neurons [15], [16], [20]. Meanwhile, binding studies on microsomal membranes from *Heliothis virescens* and *Periplaneta americana* muscles showed that diamides interact with a site different from the ryanodine binding site on the insect RyR complex. The binding site for ryanodine is located on the C-terminal portion of RyR, within or close to the pore region of the Ca^2+^ release channel [16], [20]. However, the exact binding sites for flubendiamide or chlorantraniliprole have not yet been identified.

With the wide use of diamide insecticides in the world, many studies were conducted on insect RyRs. The cDNAs encoding lepidopterous RyRs are cloned from silkworm, *Bombyx mori* (*Bm*RyR), diamondback moth, *Plutella xylostella* (*Px*RyR), beet armyworm, *Spodoptera exigua* (*Se*RyR), striped rice borer, *Chilo suppressalis* (*Cs*RyR), and rice leaf folder, *Cnaphalocrocis medinalis* (*Cm*RyR), respectively [22]–[23]. Asian corn borer, *Ostrinia furnacalis* (Guenée), is one of main maize pest insects. It was reported that diamide insecticides showed high bioefficacy against this pest insect [24]. This study aims to elucidate the structural features, developmental expression pattern and alternative splicing of *Of*RyR gene in Asian corn borers.

## Materials and Methods

### Insects and RNA preparation

Asian corn borer larvae were obtained from the stalk of field corn at the Hengshui experimental field of Chinese Academy of Agricultural Sciences, Hebei Province, China. Colonies have been maintained on an agar-free semi-artificial diet in the laboratory for several generations. Total RNA was isolated from Asian corn borer using an RNeasy® Mini Kit (Qiagen, Germany) following the manufacturer's instructions.

### *Of*RyR cDNA cloning

**Figure 1:**
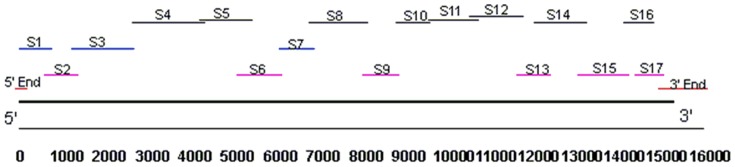
Cloning strategy for the isolation of *Of*RyR cDNA sequence. The thick black line indicates the full-length *Of*RyR cDNA. S1–S17 represent the nucleotide sequences of the overlapping cDNA clones. Six PCR fragments (S2, S6, S9, S13, S15 and S17) were amplified with degenerate primer sets. A degenerate primer coupled with a specific primer were used to obtain fragments S1, S3 and S7. And the rest fragments were amplified with specific primer sets. The 5′-end and 3′-end fragments were obtained through 5′-RACE and 3′-RACE.

Complementary DNA was transcribed from total RNA (1 µL) of third instar larvae following the manufacturer's protocol (Primescript™ First-Strand cDNA Synthesis kit; Takara, Dalian, China). The strategy used for PCR cloning is shown in Figure 1. Several primer sets based collectively on conserved sequences from *bombyx mori, Spodoptera exigua, plutella xylostella, Cnaphalocrocis medinalis*, *Heliothis virescens, Aedes aegypti, Anopheles gambiae* and *Drosophila Melanogaster* RyR mRNAs were synthesized (BGI, Beijing, China), and employed for PCR amplification (Table 1). The thermal cycling for the PCR reaction was: initial denaturing at 94°C for 5 min, 30 cycles at 94°C for 30 s, 50–65°C (depending on the Tm of primers) for 30 s and 72°C for 1–3 min (determined by the length of the amplified fragments), and an additional polymerisation step at 72 °C for 10 min. The PCR products were electrophoresed on 1% agarose gels, and the expected DNA fragments were subcloned into pMD19-T Simple Vector (Takara, Dalian, China). The nucleotide sequences of the generated clones were determined at the BGI (Beijing, China), and compared with those of other RyR mRNA sequences previously reported in GenBank.

**Table 1:** Primers used in cloning *Of*RyR cDNA.

Fragment name	Primer name	cDNA position	Primer sequence (5′-3′)
S1	F1	1–769	ATGGCBGANRSNGAGGGNRGNKC
	R1		CGGAACCGCCTTCGTAGACT
S2	F2	586–1409	TTYCAYGTRACNCAYTGGTC
	R2		TGYTTYTCYTCGTGYTCCAT
S3	F3	1237–2687	AAGTGTTCCTCGTTGTTC
	R3		ATYTTRTTYATWGCCCACAT
S4	F4	2663–4359	ACGAAATGTGGGCAATGAAC
	R4		AGACTTGCGGGACTTTGC
S5	F5	4224–5449	AGCCGAGATGTCTAAATACG
	R5		TGAGCAGGTCGTAGAAGC
S6	F6	5104–6149	MRDCCRCAYCARTGGGCTAG
	R6		TCHCKVGGHGGACANCGGAA
S7	F7	6074–6902	CAGAAATCAAGCAGTCGG
	R7		CGRCARCARGCYACVACCAT
S8	F8	6779–8160	CAGTGATGATGAACACGCTA
	R8		GAGGGTTAGCAGCCTTAGAG
S9	F9	8020–8885	CGHGARGCKGTBTCMGACTT
	R9		CKYTCVGCCATRTTYTGCAT
S10	F10	8802–9620	AGCGACTCCGTTCAACTACA
	R10		GCGTATTTACCCTCTTGC
S11	F11	9568–10731	GTTGCTGATGATTTGGGACA
	R11		AACTGTTGTCACCCGTCT
S12	F12	10513–11793	TACCCGCTGCTAATCAAG
	R12		GATGTCAATGTTGCCTCCTC
S13	F13	11614–12452	ATMCAYGARCAAGARATGGA
	R13		CCRTTDACRACRTTYCCTTC
S14	F14	12031–13252	GAATGGCAGAACTACCTCA
	R14		GCGATAAGGACGAGAATG
S15	F15	13055–14258	ARGAYGCHATHTTTGARATG
	R15		ATYTCYTTYTCNCGYTTGAA
S16	F16	14118–14837	GCACATAGACGAGGATTTC
	R16		CAGTTCTTGTTGACCTCGT
S17	F17	14374–15089	TGGGACAARTTYGYRAAGAA
	R17		ATRAARCARTTGGAYTCCATGT

Note: R = A/G, Y = C/T, M = A/C, K = G/T, S = C/G, W = A/T, H = A/C/T, B = C/G/T, V = A/C/G, D = A/G/T, N = A/C/G/T. These primers were synthetized by BGI (Beijing, China).

### Sequence analysis

The nucleotide sequences of the full-length *Of*RyR cDNA was determined by all of the overlapping cDNA clones. The molecular weight, protein length and pI were predicted using DNAMAN v.7.0 (Lynnon Corporation). The sequence alignment was performed using CLUSTALW with the default settings, and the aligned sequences were used to construct the phylogenetic tree in MEGA5. The conserved sequence was analysed using Conserved Domains (http://www.ncbi.nlm.nih.gov/Structure/cdd/wrpsb.cgi). The transmembrane (TM) segments were predicted from the secondary structure of the deduced amino acid sequence (www.cbs.dtu.dk/services/TMHMM). The hydropathy profile of the consensus amino acid sequence of *Of*RyR was analysed using the website (http://web.expasy.org/cgi- bin/protscale/ protscale.pl.).

### qRT-PCR analysis of *Of*RyR expression

The relative expression levels of *Of*RyR mRNA in different developmental stages were examined by quantitative real-time PCR (qRT-PCR). Complementary DNA was synthesized with equal amount of total RNA (1 ug) from eggs, first-instar larvae, third-instar larvae, fifth-instar larvae, pupae and adults according to the instructions of the PrimeScript™ RT reagent Kit with gDNA Eraser (Perfect Real Time) (Takara, Dalian, China). The housekeeping gene β-actin from *O. furnacalis* was utilized as a reference to normalize the amount of transcript. Primers (Table 2) for *Of*RyR and *Of*β-actin gene were designed to generate products located in the conserved domain without deletion sites. Each 20 µL PCR master mix contained 2 µL diluted cDNA template, 10 µL SYBR Premix Ex Taq™, 0.4 µL forward primer (10 uM), 0.4 µL reverse primer (10 uM), 0.4 µL Rox Reference Dye II(50×) and 6.8 µL RNase free H_2_O, which follows the instructions of the SYBR® Premix Ex Taq™ (Perfect Real Time) (Takara, Dalian, China). Thermal cycling was completed on a 7500 Real-time PCR System (Applied Biosystems) using the program of 95°C for 30 s, followed by 40 cycles of 95°C for 5 s and 60°C for 34 s. After the cycling protocol, a melting curve analysis from 60°C to 95°C was applied to all reactions to verify a single PCR product. The qPCR amplifications were carried out in 96-well plates. Four biological replicates were examined. The amplification efficiency of the target gene and housekeeping gene were estimated by using E = (10^−1/slope^)−1, where the slope was derived from the plot of the cycle threshold (Ct) value versus the log of the serially diluted template concentration. Quantification of transcript level of *Of*RyR gene was conducted according the 2^−ΔΔCt^ method [25]. The *Of*RyR expression data were expressed as means ± standard deviation (SD). Statistical analysis was performed by Duncan's Multiple Range Test for significance (P<0.05) by using SPSS 17.0 (SPSS, Inc., Chicago, IL, USA).

**Table 2:** Primers used for qRT-PCR.

Gene	Sense primer (5′-3′)	Anti-sense primer (5′-3′)	Length (bp)
*Of*RyR	GGCGGACCGAAGAATAGGGA	CGACGGCGATAAGGACGAGA	141
*Of*β-actin	ACGGCATCATCACCAACT	GGGTCATCTTTTCCCTGTT	144

### Diagnostic PCR analyses for the detection of alternative exons

Diagnostic PCR was conducted to detect the putative alternative exon in the individual cDNA clones. The names and nucleotide sequences of the primers used in the diagnostic PCR reactions were listed in Table 3. Firstly, segments containing the alternative exons were amplified by primers flanking the alternative exon region. The PCR products were cloned into pMD19-T vector (Takara, Dalian, China) and cultured overnight at 4°C. Then the recombinant plasmid was transformed into the DH5a competent cells and grew in the solid LB culture medium with ampicillin, IPTG and X-Gal. The positive clones were selected and used as templates for diagnostic PCR. Mutually exclusive exons were identified using two exon-specific primer sets. Optional exons were identified using a primer spanning the exon and flanking region or a primer spanning the flanking region excluding the exon sequence. These exon-specific primers were used to generate unique PCR products representing the presence or absence of alternative exon in each clone. The PCR products were examined by agarose gel electrophoresis and visualized under the UV transilluminator. Representative clones exhibiting unique bands were sequenced to confirm the reliability of the diagnostic PCR assay. Data were collected from 20–35 clones for each segment and developmental stage and three cDNA templates of each developmental stage were tested as replications.

**Table 3:** Primers used in diagnostic PCR.

Description	Primer name	Primer sequence (5′-3′)
Diagnostic PCR for exon a	OF1	CAGCTCTGGAAAGTGGTA
	OR1	AAAGACTCTGTGCTACCG
Diagnostic PCR for exon b	OF2	GATGCTTCCTGGATGTTT
	OR2	AACGTCTTGTCCGTATGT
Diagnostic PCR for the absence of exon c	OF3	TGGTGGTCCCGACAATCCT
	OR3	GTCCGTGCCTCCAGACTTTG
Diagnostic PCR for the presence of exon c	OF4	TGGTGGTCCCGACAATCCT
	OR4	TCACTATGTCTGGCGGGCGG

## Results

### Cloning and analysis of *Of*RyR cDNA

Totally, nineteen overlapping cDNA clones of *O. furnacalis* were amplified, and they were spliced together to make a full-length cDNA (GenBank accession number KC355370). This cDNA contains a 15327 bp open reading frame (ORF) sequence. The ORF encodes a protein of 5108 amino acid residues with molecular weight of 577.4 kDa, and the predicted isoelectric point was 5.35.

**Figure 2:**
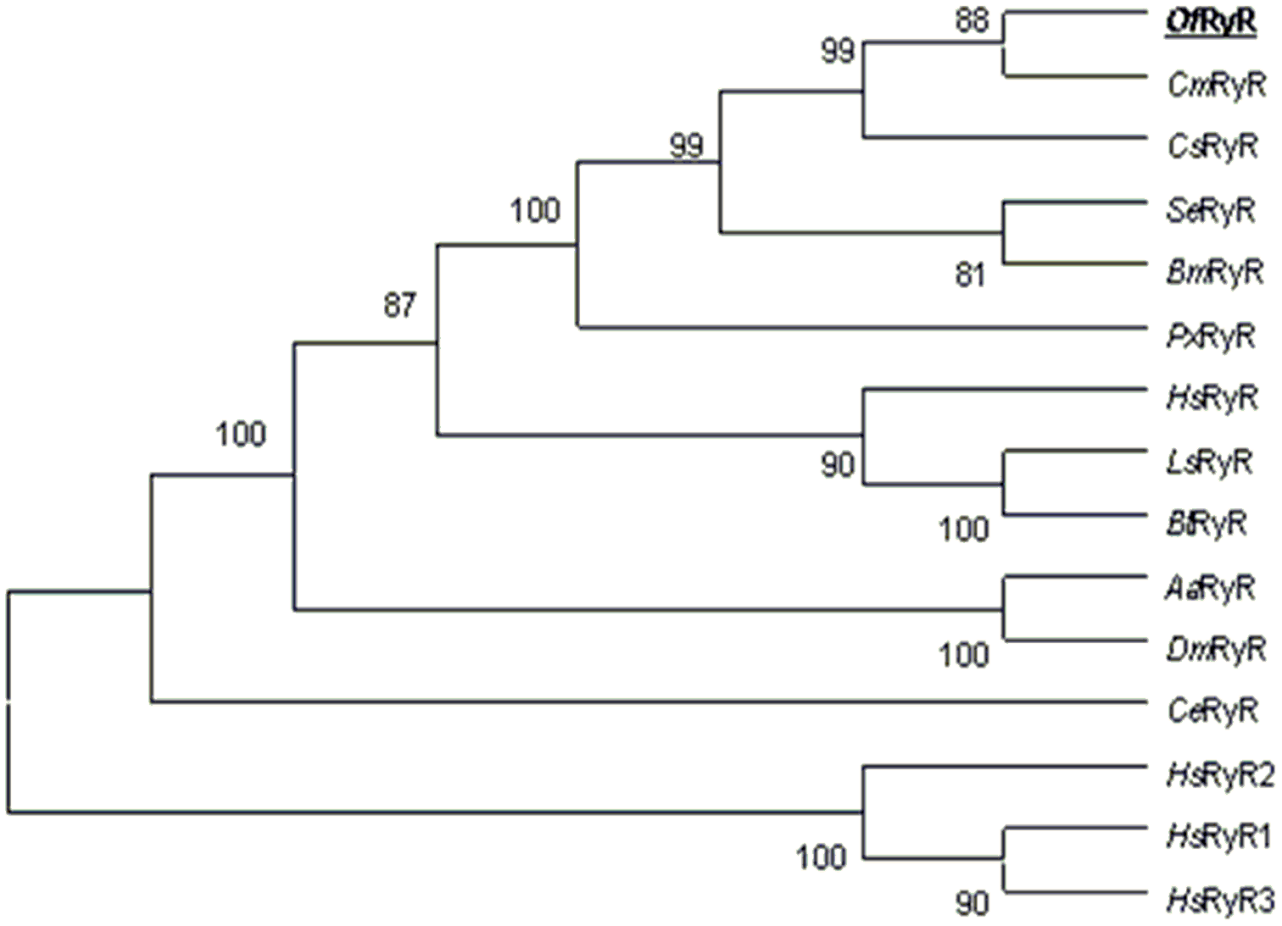
Phylogenetic tree of the RyR family. The Neighbor-joining tree was generated in MEGA5 with 1000 bootstrap replicates. The *Of*RyR amino acid sequence was aligned to 14 representative RyR isoforms and used for phylogenetic analysis. RyR sequences are obtained from GenBank: *Cnaphalocrocis medinalis*, AFI80904.1 (*Cm*RyR); *Chilo suppressalis*, AFK84958.1 (*Cs*RyR); *Spodoptera exigua*, AFC36359.1 (*Se*RyR); *Plutella xylostella*, AET09964.1 (*Px*RyR); *Bombyx mori*, DJ085056.1 (*Bm*RyR); *Harpegnathos saltator*, EFN78897.1 (*Hs*RyR); *Laodelphax striatellus*, AFK84959.1 (*Ls*RyR); *Bemisia tabaci*, AFK84957.1 (*Bt*RyR); *Aedes aegypti*, XP_001657320.1 (*Aa*RyR); *Drosophila mojavensis*, XP_002005714.1 (*Dm*RyR); *Caenorhabditis elegans*, BAA08309.1 (*C*eRyR); Homo sapiens RyR1, EAW56797.1 (*Hs*RyR1); *Homo sapiens* RyR2, EAW70071.1 (*Hs*RyR2); *Homo sapiens* RyR3, NP_001027.3 (*Hs*RyR3).

The amino acid sequences alignment showed that the identities of *Of*RyR with *Cm*RyR, *Cs*RyR, *Se*RyR, *Px*RyR, *Bm*RyR and *Dm*RyR were 97, 96, 95, 92, 95 and 79%, respectively. However, identities of *Of*RyR with *H. sapiens* RyR1–3 were only 51–60% (Table 4). To investigate the evolutionary relationships among RyR sequences, a phylogenetic analysis was performed using the ClustalW and MEGA 5.0 program based on the ORF amino acid sequence of eleven insects, one nematode and three mammalian RyRs (Fig 2). The results of phylogenetic analysis revealed that *Of*RyR is grouped with *Cm*RyR, *Cs*RyR, *Se*RyR, *Bm*RyR and *Px*RyR. The phylogenetic tree showed that the RyRs of eleven insects formed a bigger cluster, and the three mammalian RyRs formed a smaller one. This two clusters were well segregated from each other, and also from the *Caenorhabditis elegans* RyR.

**Table 4:** Percentage of amino acid sequence identity between *Of*RyR and other RyR isoforms.

	*Cm*RyR	*Cs*RyR	*Se*RyR	*Px*RyR	*Bm*RyR	*Bt*RyR	*Dm*RyR	*Hs*RyR1	*Hs*RyR2	*Hs*RyR3
*Of*RyR	97%	96%	95%	92%	95%	80%	79%	60%	52%	51%

The values correspond to the identical percentage of amino acids between the paired species. *Cnaphalocrocis medinalis* RyR (*Cm*RyR), *Chilo suppressalis* RyR (*Cs*RyR), *Spodoptera exigua* RyR (*Se*RyR), *Plutella xylostella* RyR (*Px*RyR), *Bombyx mori* RyR (*Bm*RyR), *Bemisia tabaci* RyR (*Bt*RyR), *Drosophila mojavensis* RyR (*Dm*RyR) *and Homo sapiens* RyR (*Hs*RyR)

### Primary domains of *Of*RyR

**Figure 3:**
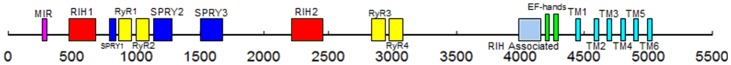
Relative position of the conserved structural domains in *Of*RyR.

**Figure 4:**
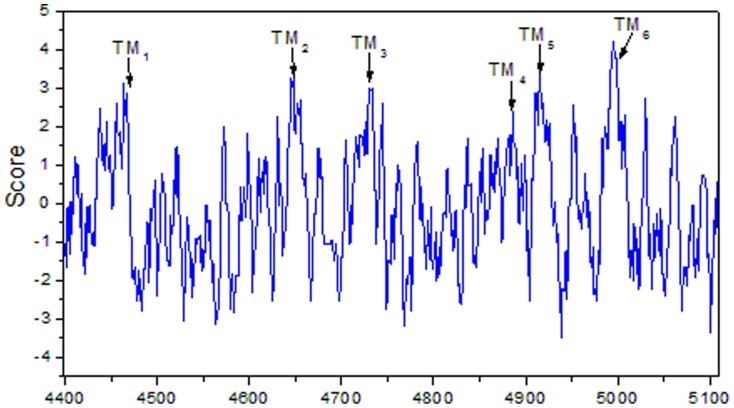
Hydropathy profile of the C-terminus *Of*RyR amino acids. The vertical axis indicates the hydropathy index, and the horizontal axis indicates the amino acid residue numbers. The positions of the predicted transmembrane segments were indicated by the arrows.

Analyses of the amino acid sequence indicated the *Of*RyR contains six hydrophobic transmembrance domains (TM1–TM6) lying between amino acids 4445 and 5007 (Table 5, Fig 3). These six sequences are amino acids Phe^4445^-Leu^4467^, Asn^4636^-Tyr^4658^, Val^4718^-Leu^4740^, Phe^4860^-Ala^4882^, Leu^4908^-Phe^4930^ and Ile^4988^-Ile^5007^. The hydropathy profile analysis further revealed that the six transmembrance domains were corresponding to six highly hydrophobic regions (Fig 4). Besides, the other conserved domains (Fig 3) in the *Of*RyR are a MIR (protein Mannosyltransferase, IP_3_R and RyR) domain at positions 276–303, two RIH (RyR and IP_3_R Homology) Domain (residues 445–654 and 2208–2435), three SPRY (SPla and RyR) domain (residues 859–953, 1097–1218 and 1528–1669), four copies of RyR (RyR repeated) domain (residues 859–953, 972–1066, 2813–2906 and 2939–3027), a RIH-associated (RyR and IP_3_R Homology associated) domain (residues 3986–4109) and two EF-hand (residues 4191–4245 and 4203–4245) domain.

**Table 5:** Positions of possible transmembrane helices.

Number	Original position	Terminal position	Length	Orientation	Sequence
TM1	4445	4467	23	Outside-inside	FYLFYYLGYGGLVVVRYIFGVLL
TM2	4636	4658	23	Inside-outside	NFYNLKYVALVLAFCINFVLLFY
TM3	4718	4740	23	Outside-inside	VINMAAVLHSIVSLAILIGYYHL
TM4	4860	4882	23	Inside-outside	FLYSLWYFSFSVMGNFNNFFFAA
TM5	4908	4930	23	Outside-inside	LVLTVMLLTIIVYIYTVIAFNFF
TM6	4988	5007	20	Inside-outside	ISFFFFIIVILLAILQGLII

### Analysis of *Of*RyR amino acid sequence

Important modulator-binding sites were identified in *Of*RyR amino acid sequence (Fig S1) when compared to other reported RyRs including *Bm*RyR, *Px*RyR, *Cm*RyR, *Dm*RyR, *Hv*RyR (*Heliothis virescens* RyR) and *Oc*RyR1 (*Oryctolagus cuniculus* RyR). A consensus sequence, Y[GAST][VG][KTQSN], for the putative adenine ring binding domain [26], was found in three sites (residues 1093–1096, 1145–1148 and 5073–5076) of *Of*RyR sequence. Three possible nucleotide binding sites, identified on the basis of the consensus GXGXXG motif [27], were located at positions 2734–2739, 3976–3981 and 4687–4691 in the *Of*RyR sequence. A potential binding site of calmodulin (CaM) in *Of*RyR was recognised in residues 3722–3751, corresponding to residues 3614–3643 in rabbit RyR1 sequence [28]. Two putative Ca^2+^-binding EF-hand motifs (residues 4186–4213 and 4221–4248), which interact with the cytoplasmic Ca^2+^ to regulate the ion channel were present in *Of*RyR. It has been shown that C-terminal region of RyR could form the functionally important domains. For example, the pore helix was predicted to be in the loop region between the fifth and sixth transmembrane helices (residues 4949–4963) [13]. Covering part of this sequence (residues 4960–4969), the sequence motif (GVRAGGGIGD), which has been shown to constitute pore-forming segments of the Ca^2+^ release channels was present in *Of*RyR [22]. All of these residues are also conserved in the *Of*RyR sequence as well as in other RyRs sequence. Moreover, a glutamate that is proposed to be involved in the Ca^2+^ sensitivity in rabbit RyR1 (E^4032^) [29] was conserved in *Of*RyR (E^4145^). Residues corresponding to I^4897^, R^4913^, and D^4917^ of rabbit RyR1, which play an important role in activity and conductance of the Ca^2+^ release channel [30], were also conserved in *Of*RyR (I^4967^, R^4983^, and D^4987^).

### Expression of *Of*RyR mRNA in different developmental stages

**Figure 5:**
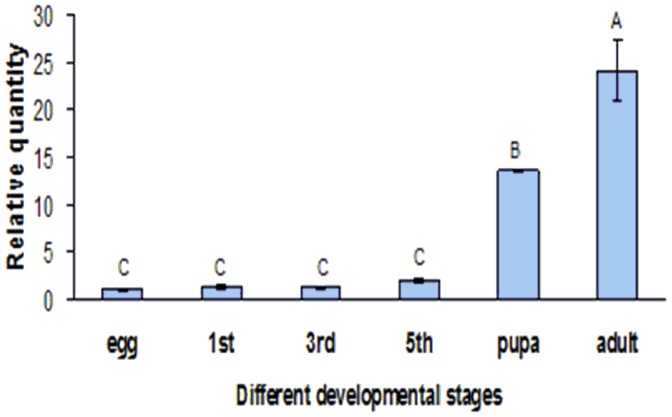
The relative expression levels of *Of*RyR in different developmental stages of *O. furnacalis*. Data were presented as means ± SE for four independent replicates. 1st, 3rd and 5th denotes first-instar, third-instar and fifth-instar larva. Different letters above each bar indicate statistical difference by ANOVA followed by the Duncan's Multiple Comparison test (P<0.05).

The amplification efficiency (E) values of RyR and β-actin from *O. furnacalis* were calculated to be 0.925 and 0.963 respectively. Moreover, the relative expression level of *Of*RyR showed a significant difference between different developmental stages (Fig 5). It showed the lowest expression level in egg and the highest in adult, and the relative expression level in adult and pupae were 24.1 and 13.6 times when compared to that in egg. In addition, the expression level of *Of*RyR in first, third and fifth-instar larvae were 1.28, 1.19 and 1.99 times of that in egg.

### Developmental regulation of alternative exon usage

**Figure 6:**
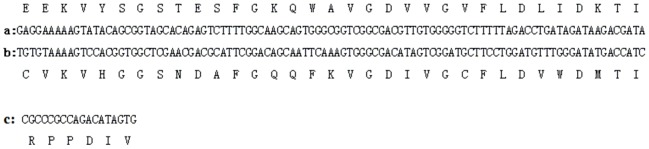
Nucleotide and putative amino acid sequences of alternative exons in the *Of*RyR gene.

Multiple sequences alignment revealed two putative alternative splicing sites (named alternative splicing 1 (AS1) and alternative splicing 2 (AS2)) existed in *Of*RyR. This two splicing sites form three alternative exons. Figure 6 showed the nucleotide and putative amino acid sequences of the three alternative exons. AS1 located between amino acid residues 1141–1173, which corresponding to the central part of the predicted second SPRY domain and forms one pair of mutually exclusive exons (a/b). AS2 located between the predicted third and fourth RyR domains (residues 2926–2931) and forms the optional exon c.

**Figure 7:**
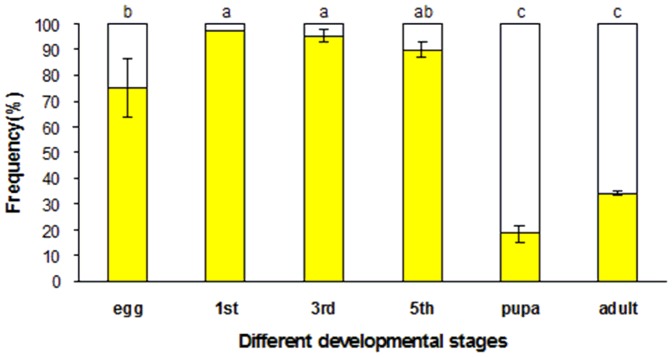
The usage frequencies of *Of*RyR alternative exons (a and b) in different developmental stages of O. furnacalis. Mutually exclusive exons were represented by yellow bars (exon a) and open bars (exon b). 1st, 3rd and 5th denotes first-instar, third-instar and fifth-instar larva. Different letters above each bar indicate statistical difference by ANOVA followed by the Duncan's Multiple Comparison test (P<0.05).

**Figure 8:**
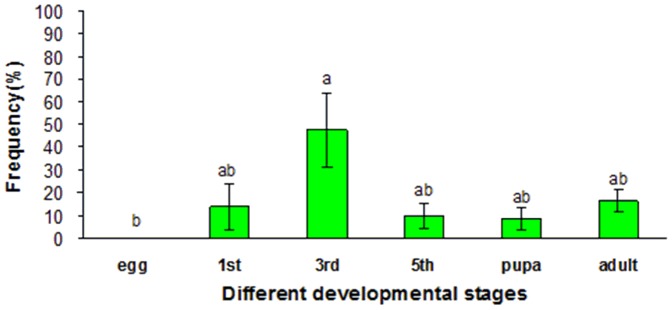
The usage frequencies of *Of*RyR optional exon c in different developmental stages of *O. furnacalis*. Optional exon c was indicated by green bars. 1st, 3rd and 5th denotes first-instar, third-instar and fifth-instar larva. Different letters above each bar indicated statistical difference by ANOVA followed by the Duncan's Multiple Comparison test (P<0.05).

Diagnostic PCR was used to determine the usage of each alternative exon for *Of*RyR mRNA at different developmental stages including egg, first instar larva, third instar larva, fifth instar larva, pupae and adult. The usage frequencies of each putative alternative exon were summarized in Figure 7 and 8. The usage of the mutually exclusive exons (a/b) exhibited significantly developmental regulation. Exons a and b existed in all developmental stages of *Of*RyR cDNA clones but showed different level of usage frequency. In the first instar, third instar and fifth instar larva cDNA, the usage frequency of exon a was 97±0.1%, 95.2±2.6% and 89.8±2.8%. In egg cDNA, it amounted to 75.2±11.2%. But it was only 18.4±3.4% and 34.1±0.7% in pupae and adult cDNA. As for exon c, the highest frequency, 47.5±16.3%, was observed in the third instar larvae cDNA, and the relatively low frequency was found in the first instar larva, fifth instar larva, pupae and adult cDNA, which amounted to 13.6±10.1%, 9.6±5.7, 8.3±4.7% and 16.1±4.9%, respectively. However, exon c was not detected in egg cDNA.

## Discussion

Although insect RyR has been used as target of insecticide for more than six decades [31], little research was conducted upon it until the emergency of diamides insecticides. These insecticides show highly insecticidal activity against lepidopteran insect pests but exhibit low mammalian toxicity. For this reason, insects RyRs have been studied with increasing interest in the pesticide field. Recently, XL Wang et al. reported a *Px*RyR that is composed of 5164 amino acids [22]. L Guo et al. cloned two *Px*RyR cDNA, *Px*RyR1 and *Px*RyR2 [31], they are respectively 47 and 91 amino acids shorter than the *Px*RyR cloned by Wang et al. And LN Sun et al. also reported a *Px*RyR showing 99% amino acids identity with Wang's RyR [23]. In addition, the *Cm*RyR cDNA encoding 5087 amino acid residues was cloned by JJ Wang et al. [13]. In our study, we cloned and sequenced the full-length cDNA encoding *Of*RyR from third instar larva of *O. furnacalis*. The deduced 5108 amino acid residues of *Of*RyR shares a high degree of identity (92%–97%) with reported lepidopteran insect RyRs. But *Of*RyR shares only 48–60% identity with mammalian isoforms. The amino acid divergence between insect and mammalian RyR isoforms might be a reason why diamide insecticides show a high selectivity for insect RyRs compared to mammalian RyRs. Moreover, seven residues (N^4922^, N^4924^, N^4935^, L^4950^, L^4981^, N^5013^ and T^5064^), which are unique to lepidopteran RyR homologues have been found in *Cm*RyR sequence [13]. The identical residues (N^4943^, N^4945^, N^4956^, L^4971^, L^5002^, N^5034^ and T^5085^) were also identified in *Of*RyR sequence. In contrast, different residues at the corresponding positions are shared by non-lepidopteran insect RyRs. And these residues are highly conserved in the non-lepidopteran insects, other invertebrate and vertebrate RyRs [13]. Interestingly, the residue N^4956^ in *Of*RyR just locate in the central part of the putative pore-helix motif, and the residue L^5002^ is present in the sixth transmembrance domain. These results indicated that these residues might be involved in the differences in Ca^2+^ release channel properties between lepidopteran and non-lepidopteran insects RyRs.

The diamide insecticides are potent activators of insect RyRs. It has been proved that the action of flubendiamide is highly specific, with selective toxicity in restricted insect taxa, including Lepidoptera [15]. For example, flubendiamide could induce release of Ca^2+^ through the *Bm*RyR, but not through the rabbit RyR isoforms [9]. However, the binding sites of diamide insecticides are not clear. Binding studies on microsomal membranes from insect muscles suggest that flubendiamide and chlorantraniliprole interact with a site distinct from the ryanodine binding site on the insect RyR complex [16], [20]. And flubendiamide is mainly incorporated into the transmembrane domain (amino acids 4111–5084) of the sRyR, while the N-terminal sequence (residues 183–290) is a structural requirement for flubendiamide-induced activation of the sRyR [9]. This segment of sRyR (residues 183–290) shares 94–99% identity with the equivalent region of other insects RyRs (*Of*RyR, *Cm*RyR, *Se*RyR and *Px*RyR), but only 49% identity between sRyR and rabbit RyR1. This is possibly an important reason why diamide insecticides show high selectivity between pest insects and mammals.

Adenine nucleotides (including ATP, ADP, AMP, cAMP, adenosine, and adenine) are also activators of RyRs. Studies using photoreactive ATP analog Bz2ATP indicated the regulation of Ca^2+^ release by ATP involves an ATP binding site(s) located on the 27-kDa and 13-kDa fragments of the ryanodine receptor protein [22], [32]. The consensus sequence for an ATP-binding site, GXGXXG, occurs between three and six times in the RyR sequence. And there were three possible nucleotide binding sites, located at positions 2734–2739, 3976–3981 and 4687–4691 in the *Of*RyR sequence. In addition, *Of*RyR might be regulated by cytosolic Ca^2+^, similar to mammalian RyRs [33]. Because two conserved Ca^2+^-binding EF-hands were present in the C-terminus of the *Of*RyR. Ca^2+^ is the principal regulation mechanism for RyR in excitation-contraction coupling. And RyR was activated by micromolar concentrations of cytoplasmic Ca^2+^ [3], but millimolar Ca^2+^could inhibit RyR. This biphasic behavior may result from two classes of Ca^2+^ binding sites, a high-affinity activation site and a low affinity inactivation site. Another important regulator of *Of*RyR is the bifunctional protein calmodulin (CaM). The mammalian RyRs are regulated by CaM both in its Ca^2+^-free CaM (apo-CaM) and Ca^2+^-bound CaM (Ca^2+^-CaM) states [34]. When free Ca^2+^ is between 10^−9^ and 10^−7^ mol/L, apo-CaM activates RyR1 and RyR3 (but not RyR2), whereas the concentration of Ca^2+^ >10^−5^ mol/L, Ca^2+^-CaM inhibits all three RyR isoforms [35]. A potential binding site for both apo-CaM and Ca^2+^-CaM was identified in the *Of*RyR sequence (residues 3722–3751) [28]. So this region in *Of*RyR sequence might be the candidates for CaM binding and play a crucial role in the physiology process.

Real-time quantitative PCR indicated the expression level of *Of*RyR gene was quite different in the development stages. It showed high expression level in adult and pupae and the lowest in egg. However, Guo et al have reported that the expression levels of the *Px*RyR in different-instar larvae and adults were much higher than those of the prepupae and pupae in *P. xylostella* [31]. Therefore, we could deduce that the expression level of RyRs might vary in different insects.

Alternative splicing of pre-mRNA transcripts is a prevalent feature of gene processing and powerful mechanism to generate remarkable protein diversity from single gene [36]. And it plays an important role in physiology, disease and developmental stage-specific processes [37], [38]. Recently, many alternative splice variants of RyR isoforms have been identified in human, rabbit and mouse. For instance, alternative splicing of ASI residues (A^3481^–Q^3485^) contribute to an inhibitory module in RyR1 that influences excitation-contraction coupling [39]. And ASI residues is developmentally regulated: the residues are present in adult ASI(+)RyR1, but absent in the juvenile ASI(−)RyR1 which is over-expressed in adult myotonic dystrophy type 1 [1]. The 24-bp splice insertion of human RyR2, which is present at low level in embryonic and adult hearts can suppress intracellular Ca^2+^ fluxes that protects cells from caffeine-evoked apoptosis [36]. And a short isoform of spliced RyR3, which lacks a 29 amino acid fragment (H^4406^–K^4434^) containing a predicted transmembrane helix, is not a functional Ca^2+^ channel but inhibits the efficiency of the Ca^2+^-induced Ca^2+^ release mechanism by a negative modulation of RyR2 in native smooth muscle cells [36].

Similarly, alternative splicing also exists in insects RyRs. It’s reported that the multiple mRNAs of *D. melanogaster* RyR can be generated by two different splicing patterns: (i) the mutually exclusive incorporation of exon into mRNA, and (ii) the choice of one out of two splice-acceptor sites [20]. The same splicing patterns were also identified in the *C. medinalis* and *O. furnacalis* RyR genes. In our study, two alternative splice variants (AS1 and AS2) showing developmental regulation were found in the *Of*RyR gene. AS1 (residues 1141–1173) located in the central of the second SPRY domain (SPRY2). The SPRY domain is a protein-protein interaction module and is implicated in important biological pathways, including regulate innate and adaptive immunity [40]. SPRY2 has been identified as an in vitro binding partner for N-terminal residues in the II–III loop of the skeletal muscle DHPR, as well as for the scorpion toxins imperatoxin A and maurocalcine. In addition, SPRY2 domain and its F loop (P^1107^-A^1121^) bind to the alternatively spliced residues and neighboring basic residues (ASI/basic) of RyR1 via an electrostatic interaction to regulate excitation contraction coupling. Mutation of the negatively charged amino acid of the SPRY2 F loop reduce SRRY2 domain binding to the ASI/basic region [41]. Since the mutually exclusive exons a and b are highly different in *Of*RyR, and exon b contains less negatively charged amino acids than exon a. Therefore, the *Of*RyR splice variants generated by mutually exclusive exons a and b might probably possess different function and binding effect. In our study, the splice variants showed significantly developmental regulation in *Of*RyR. This result implied that alternative splicing might play an important part in the temporal encoding of the Ca^2+^ release channel, and might involve in the differences in channel properties and function.

## Supporting Information

Figure S1 **Analysis of the deduced** ***Of*** **RyR amino acid sequences.** Predicted transmembrance domains were overlined and labeled TM1–TM6. Conserved residues for the putative EF-hand motifs, pore-forming segments, pore-helix, adenine ring binding sites (Y[GAST][VG][KTQSN]), nucleotide binding sites (GXGXXG) and potential CaM binding site were also overlined and labeled. The residues in *Of*RyR (I4967, R4983 and D4987), which play an important role in activity and conductance of the Ca2+ release channel were marked with triangles. Pentastar indicated glutamate (E4145) that is proposed to be involved in the Ca2+ sensitivity in *Of*RyR.
(DOC)
