# Supplementary material for: Molecular Cloning, Characterization and Expression Profiling of a Ryanodine Receptor Gene in Asian Corn Borer, Ostrinia furnacalis (Guenée)
Source: PLoS One. 2013 Oct 1;8(10):e75825. doi: 10.1371/journal.pone.0075825 (PMC3787966; doi:10.1371/journal.pone.0075825)
Supplement: Figure S1 — Analysis of the deduced Of RyR amino acid sequences. Predicted transmembrance domains were overlined and labeled TM1–TM6. Conserved residues for the putative EF-hand motifs, pore-forming segments, pore-helix, adenine ring binding sites (Y[GAST][VG][KTQSN]), nucleotide binding sites (GXGXXG) and potential CaM binding site were also overlined and labeled. The residues in OfRyR (I4967, R4983 and D4987), which play an important role in activity and conductance of the Ca2+ release channel were marked with triangles. Pentastar indicated glutamate (E4145) that is proposed to be involved in the Ca2+ sensitivity in OfRyR. (DOC) [file pone.0075825.s001.doc]

1 MADGEGGASE QDDVSFLRTE DMVCLSCTAT GERVCLAAEG FGNRHCFLEN IADKNIPPDL SQCVFVIEQA

71 LSVRALQELV TAAGSETGKE NLGKGTGSGH RTLLYGNAIL LRHLNSDMYL ACLSTSSSQD KLAFDVGLQE

141 HSQGEACWWT LHPASKQRSE GEKVRVGDDL ILVSVATERY LHTTKENEVS IVNASFHVTH WSVQPYGTGI

211 SRMKYVGYVF GGDVLRFFHG GDECLTIPSA WAKETGQNIV VYEGGSVMSQ ARSLWRLELA RTKWAGGFIN

281 WYHPMRIRHI TTGRYLGVND QNELYLVSRE EATTASCAFC LRQEKDDQKV VLEDKDLEVI GAPIIKYGDS

351 TVIVQHSETG LWLSYKSYET KKKGVGKVEE KQAILHEEGK MDDGLDFSRS QEEESRTARV IRKCSSLFTK

421 FINGLETLQE NRRHSMFFAS VNLGEMVMCL EDLINYFAQP DEDMEHEEKQ NKFRALRNRQ DLFQEEGILN

491 LILEAIDKIN VITSQGFLAG FLAGDESGQS WDMISGYLYQ LLAAIIKGNH TNCAQFANSN RLNWLFSRLG

561 SQASGEGTGM LDVLHCVLID SPEALNMMRD EHIKVIISLL EKHGRDPKVL DVLCSLCVGN GVAVRSSQNN

631 ICDYLLPGKN LLLQTQLVDH VSSVRPNIFV GRVEGSAVYQ KWYFEVTMDH IEKTTHMMPH LRIGWANTAG

701 YVPYPGGGEK WGGNGVGDDL YSFGFDGAFM WSGGRKTPVN RTHAEEPYIR KSDVIGCALD LTVPIINFMF

771 NGVRVTGSFT NFNLEGMFFP VISCSSKLSC RFLLGGEHGR LRYAAPEGYS PLVESLLPQQ ILALEPCFYF

841 GNLAKRALAG PPLVQDDTAF VPTPVDTLQI TLPSYVEQIR DKLAENIHEM WAMNKIEAGW MYGDQREDLH

911 KIHPCLVPFE RLPAAEKRYD IQLAVQTLKT ILALGYYISL DKPPARIRNV RLPNEPFMQS NGYKPAPLDL

981 SAVTLTPKMD ELVDQLAENT HNLWARERIQ QGWTYGLNED PDMHRSPHLV PYPKVDDAIK KANRDTASET

Adenine ring binding 1

1051 VRTLLVYGYN LDPPTGEQHE ALLAEASKQK QADFRTYRAE KNYAVSSGKW YFEFEILTAG PMRVGWAHAD

Adenine ring binding 2

1121 MAPGMMLGQD ENSWAFDGYN EEKVYSGSTE SFGKQWAVGD VVGVFLDLID KTISFSLNGE LLMDALGGET

1191 TFADVQGDNF VPACTLGVGQ KARLTYGQDV NTLKYFTTCG LQEGYEPFCV NMKRDVTHWY TKDQPIFENT

1261 DEMLDTRIDV TRIPAGSETP PCMKISHNTF ETMEKANWEF LRLSLPVICQ AQFIGEQEKA RRWVEIKERQ

1331 QILMKEATEA QMPAHIDQIM RSGFTMNDIK GLHYEDNQDD VQSKMKRQPS RPPRKGSVTR GVTYQQGQVN

1401 GMHRSTSEAE MSKYELGVQN LADEKKDKRG RSPFKFFKSK RGESGDRAKS RKSKTPDPFS DTEVSPERGP

1471 RRPNPQIRVS QTDNQQTNLQ MATPTQDRKQ MTTSALSAAT TETVGNEIFD AECLKLINEY FYGVRIFPGQ

1541 DPTHVYIGWV TTQYHLHSKD FNQNKVMKSS VIITDDYDRV VESVNRQSCY MVRADELYNE VMAEATGPKG

1611 ASQGMFIGCS VDTSTGTVAF TCEGKDTSIK FKMEPETKLF PAIFVEATSK EILQIELGRS STSLPLSAAV

1681 LPTSDKHVIP QFPPRLKVQC LKPHQWARVP NQSLQVHALK LSDIRGWSML CEDAVSMLAL HIPEEDRCID

1751 ILELIEMDKL LSFHSHTLTL YAALCYQSNY RAAHALCQHV DQKQLLYAIK SQYMSGPLRQ GFYDLLIALH

1821 LESHATTMET CKNEFVIPLG PELKALYDEA GMGHSLRSLQ TESVRPQMKM TDIAENISDI SNLYSPYFPL

1891 EVVREFLMQA LAEAVETNQV HNRDPVGGSN ENLFLPLIKL VDRLLLVGMM RDEDVEKLLI MINPETWDPT

1961 FDREGKDEHR KGLLHMKMAE GAKLQMCYLL QHLNDIQLRH RVESIIAFAH DFVGDVQTDQ LRRYTEIKQS

2031 DLPSAVAAKK TREFRCPPRE QMNAILSFKH LEEEDKENCP CGEDLIARMN EFHESLMSHV SLNALQEPDP

2101 GENAEPEAKP GAFGKLYNII NTVKELEEEP KAIEEPPKKT PEEKFRKVLI QTIVSWAEES QIETPKLVRE

2171 MFSLLVRQYD AVGELIRALE KTYVINAKTK LDVAEMWVGL SQIRALLPVQ MSQEEEELMR KRLWKLVNNH

2241 TFFQHPDLIR VLRVHENVMA VMMNTLGRRA QAQSDAQPAS PPTADDKEKD TSHEMVVACC RFLCYFCRTG

2311 RQNQKAMFDH FDFLLENSNI LLSRPSLRGS TPLDVAYSSL MENTELALAL REHYLEKIAV YLSRCGLQSN

2381 SELIEKGYPD LGWDPVEGER YLDFLRFCVW VNGESVEENA NLVIRLLIRR PECLGPALRG EGEGLLKAIV

2451 DANKMSERIA DRRKLREMEQ EGDVTFSHPL PESDEDEDYI DTGAAILNFY CTLVDLLGRC APDAAVIALG

2521 KNESLRARAI LRSLVPLEDL QGVLSLRFTL NNPAAGEERP KSDMPSGLIP GHKQSVGLFL ERVYGIETQE

2591 LFYRLLEEAF LPDLRAATML DRNDGCESDM ALSMNRYIGN SILPLLIKHA NFYNEAENYA SLLDATLHTV

2661 YRLSKNRMLT KGQREAVSDF LVALTSAMQP SMLLKLLRKL TVDVSRLSEY TTVALRLLTL HYERCAKYYG

Nucleotide binding 1

2731 STGGQGIYGA SSDEEKRLTM MLFSNIFDSL SKMDYEPELF GKALPCLIAI GCALPPDYSL SKNYDDEFYG

2801 KETQATGGPD NPQYDPQPIN TSSVALNNDL NTIVQKFSEH YHDAWASRKI ENGWVYGESW SDSQKAHPRL

2871 KPYNMLNDYE KERYKEPVRE SLKALLAIGW SVEHSEVDIP STNRSSMRRQ SKSGGRPPDI VTDSATPFNY

2941 NPHPVDMTNL TLSREMQNMA ERLAENAHDI WAKKKKEELV TNGGGIHPQL VPYDLLTDKE KKKDRERSQE

3011 FLKYLQYQGY KLHRPSKSSP SDTEQTTTGV AIELRFAYSL LEKLIQYIDR ATINMKLLKP STTFSRRSSF

3081 KTSTRDIKFF SKVVLPLMEK YFSTHRNYFI AVATATNNVG AASLKEKEMV AALFCKLASL LRSRLAAFGP

3151 DVRITVRCLQ VLVKGIDAKS LVKNCPEFIR TSMLTFFNNV ADDLGHTILN LQEGKYAHLR GTHLKTSTSL

3221 GYINGVVLPI LTAKFDHLAN CEYGADLLLD EIQVASYKML GSLYALGTDA TLTHDRKYLK TEIERNKPAL

3291 GSCLGAFSST FPVAFLEPHL NKHNQFSLLN RIADHSLEAQ DIMAKMEQSM PTLETILSEV DQFVESDKTY

3361 NEAPHIIDVV LPLLCSYLPF WWAQGPDNVT PTGGNHVTMV TAEHMNQLLK NVLKLIKKNI GNESAPWMTR

3431 IATYTQQIII NSSEELLRDS FLPLAERVRK RTDTMFHKEE SLRGFIKSST DDTSQVESQI QEDWQLLVRD

3501 IYSFYPLLIK YVDLQRNHWL RNNVPEAEEL YNHVAEIFNI WSKSQYFLKE EQNFISANEI DNMVLIMPTA

3571 TRRVTTVVEG APQGGGKKKK KHRDKKRDKD KEVQASLMVA CLKRLLPVGL NLFAGREQEL VQHCKDRFLK

3641 KMTEQDVAEF AKTQLTLPDK IDPADEMSWQ HYLYSKLGSK SKANITADTA ENKAKIIDDT VERIVAMSKV

CaM binding

3711 LFGLHMIDHP QQMSKNVYRS VVSIQRKRAV IACFRQLSLH SLTRHRACNI FARTYYELWL EEENVGQEVM

3781 IEDLTQSFED AELKKSDVVE EEGKPDPLTQ LVTTFCRGAM TERSGALQED LLYMSYANII AKSCGEEEEE

3851 GGGEEEEGGG EAEGEEEGRA SIHEQEMEKQ KLLFHQARLA DRGVAEMVLL HISASKGVPS EMVMKTLELG

Nucleotide binding 2

3921 ISILRGGNID IQMGMLNHLK DKKDVGFFTS IAGLMNSCSV LDLDAFERNT KAEGLGVGLE GAAGEKNMHD

3991 AEFTCALFRF IQLTCEGHNL EWQNYLRTQA GNTTTVNVVI CTVDYLLRLQ ESIMDFYWHY SSKELIDPAG

4061 KANFFKAIGV ASQVFNTLTE VIQGPCTQNQ QALAHSRLWD AVGGFLFLFS HMQDKLSKHS SQVDLLKELL

EF-hand1

4131 NLQKDMITMM LSMLGNVVN GTIGKQMVDT LVESASNVEL ILKYFDMFLK LKDLTSSASF QEIDANNDGW

EF-hand2

4201 VLPKDFKEKM EQQKSYTPEE IEFLLACRET NHDGKLDYIG FCDRFHEPAK EIGFNLAVLL TNLSEHMPNE

4271 PRLARFLETA GSVLNYFEPF LGRIEIMGGS KRIERVYFEI KESNIEQWEK PQIKESKRAF FYSIVTEGGD

4341 KEKLEAFVNF CEDAIFEMTH ASSLMAACEE SAGGPKNREA SYMYMGDDDD ERAGKDPFRR GLQSVKDGIS

TM1

4411 TAFSSLSPSN IKQKIADLQQ MPPAELAVGF FKMFFYLFYY LGYGGLVVVR YIFGVLLGLM RGPQMEEPPP

4481 EPTEEEKIGQ LRHRLLAQSP SRHLPALPPA DDTGQMQVQA FGLDITKEDN GQIQVKPHES PSTSSPSSGE

4551 EADASLDEGA EHTEEQRPPS LIDLLGGEQA KKQAQERMEA QAVQQAAMSA IEAESKKAVQ VPAPSALAQV

TM2 Nucleotide binding 3

4621 DLSQYTRRAV SFLARNFYNL KYVALVLAFC INFVLLFYKV SALDSEEGEG SGLGDIIGGS GSGQGSGSGD

TM3

4691 GGSGESGEDE DALEVVHIDE DFFYMVHVIN MAAVLHSIVS LAILIGYYHL KVPLAIFKRE KEIARKLEFD

4761 GLYIAEQPED DDLKSHWDRL VISAKSFPVN YWDKFVKKKV RAKYSETYDF DSISNMLGME KTSFSAQEEE

TM4

4831 GSKGLIHYIM NIDWRYQVWK AGVTITDNSF LYSLWYFSFS VMGNFNNFFF AAHLLDVAVG FKTLRTILQS

TM5 Pore-helix Pore-forming segment

4901 VTHNGKQLVL TVMLLTIIVY IYTVIAFNFF RKFYVQEEDD EVNKNCHDML TCFVFNLYKG VRAGGGIGDE

TM6

4971 LEPPDGDDSE VYRIIFDISF FFFIIVILLA ILQGLIIDAF GELRDQLESV KEDMESNCFI CGINKDYFDK

Adenine ring binding 3

5041 VPHGFDTHVQ REHNLANYMF FLMHLINKPD TEYTGQETYV WNMYTQRCWD FFPVGDCFRK QYEDVMGE*

**Figure S1. Analysis of the deduced *Of*RyR amino acid sequences.**
